# Effect of etching time on morphological, optical, and electronic properties of silicon nanowires

**DOI:** 10.1186/1556-276X-7-393

**Published:** 2012-07-16

**Authors:** Nesma Nafie, Manel Abouda Lachiheb, Mongi Bouaicha

**Affiliations:** 1 Laboratoire de Photovoltaique, Centre de Recherches et des Technologies de l’Energie, Technopole de Borj-Cedria, BP 95, Hammam-Lif, Tunis, 2050, Tunisia

**Keywords:** Silicon nanowires, Reflectivity, Light-beam-induced-current, Diffusion length

## Abstract

Owing to their interesting electronic, mechanical, optical, and transport properties, silicon nanowires (SiNWs) have attracted much attention, giving opportunities to several potential applications in nanoscale electronic, optoelectronic devices, and silicon solar cells. For photovoltaic application, a superficial film of SiNWs could be used as an efficient antireflection coating. In this work we investigate the morphological, optical, and electronic properties of SiNWs fabricated at different etching times. Characterizations of the formed SiNWs films were performed using a scanning electron microscope, ultraviolet–visible-near-infrared spectroscopy, and light-beam-induced-current technique. The latter technique was used to determine the effective diffusion length in SiNWs films. From these investigations, we deduce that the homogeneity of the SiNWs film plays a key role on the electronic properties.

## Background

Silicon nanowires (SiNWs) have attracted much attention in the recent years due to their importance in the field of electronic devices and photovoltaic [1-4]. Hence, SiNWs could be used as an antireflection coating due to the reduction of optical loss which is an important factor to obtain efficient Si solar cells. However, when SiNWs are used as an antireflection coating, a great care should be taken to avoid degradation of the electronic properties, which in turn can increase the serial resistance of the solar cell. Different methods have been employed to fabricate SiNWs, such as chemical physical deposition [5], laser ablation [6,7], thermal evaporation [8,9], and etching. In this paper, we used the silver-assisted chemical etching technique [10-15]. We fabricate SiNWs at different durations, ranging from 10 to 90 min.

## Methods

Substrates used in this study are P^+^ silicon wafers, boron-doped and (100) oriented, with thickness of 500 μm and resistivity of 0.01 to 0.02 Ωcm. After cleaning, silicon samples were immersed into the etching solution containing 0.05 M AgNO_3,_ 40% HF, and H_2_O_2_ at room temperature for different etching times; 10, 20, 30, 40, 50, 60, 70, 80, and 90 min. After etching, samples were rinsed with deionized water to remove residual HF and immersed in a H_2_O-HNO_3_ (2 and 1 V) solution during several seconds to remove the silver film.

The morphology of samples was analyzed using a scanning electron microscope (SEM). We performed top and cross-section SEM images of the samples. The cross-section SEM images were used to evaluate the length of SiNWs. We measured the surface reflectivity in the 250 to 1,250 nm spectral range by a UV–vis-NIR spectrophotometer. To study the electronic properties of the formed films, we evaluate the effective diffusion lengths (*L*) of minority carriers in the SiNWs films. Values of *L* were carried out from the light-beam-induced-current (LBIC) profiles measured on metal/SiO_2_/SiNWs/c-Si/metal diode.

## Results and discussion

**Figure 1:**
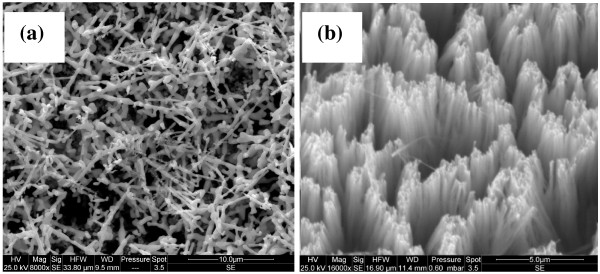
** Top SEM views of one sample.** (**a**) Before, and (**b**) after removing the silver film.

**Figure 2:**
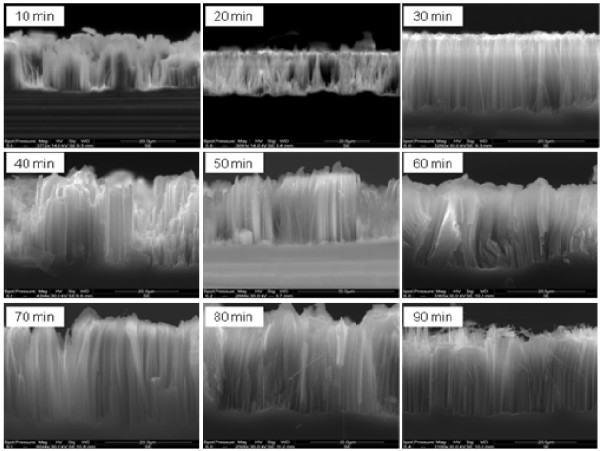
Cross-section SEM images of formed SiNWs at different durations.

**Figure 3:**
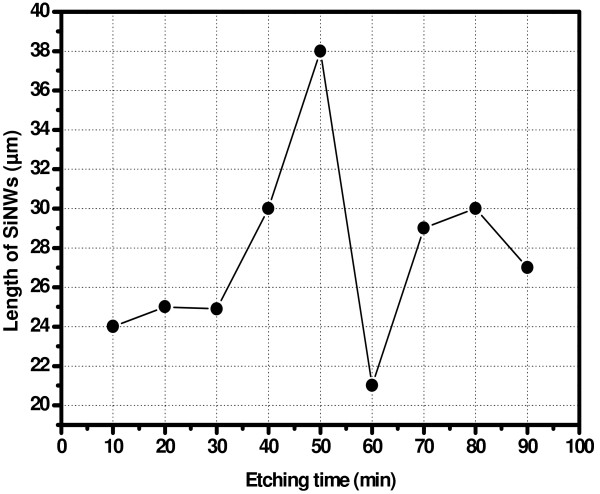
Variation of the length of SiNWs films vs. etching time.

In Figure 1 we give the top SEM views of one sample, before (Figure 1a) and after (Figure 1b) removing the silver film. One can see in Figure 1a, the silver dendrites formed during the etching process. In Figure 1b, we give a tilted SEM view of the SiNWs after removing the Ag film. In Figure 2, we give cross-sectional SEM images of SiNWs films prepared at 10, 20, 30, 40, 50, 60, 70, 80, and 90 min etching times. As reported in Figure 3, the mean length of the film varies from 21 to 38 μm. From Figure 3, we notice that the etching velocity is similar for 10, 20, and 30 min. However, it is more important for 40 and 50 min, where the maximum length is reached. When the sample is etched at greater durations, the length decreases by 10 μm from its maximum value and seems to be stabilized at values around 28 μm. This was attributed to the fact that when the etching process started, the HF solution etches the silicon substrate leading to an increase of the SiNWs’ length. After reaching the maximum length at 50 min, SiNWs themselves are etching by the HF solution as observed in the SEM image corresponding to 60 min.

**Figure 4:**
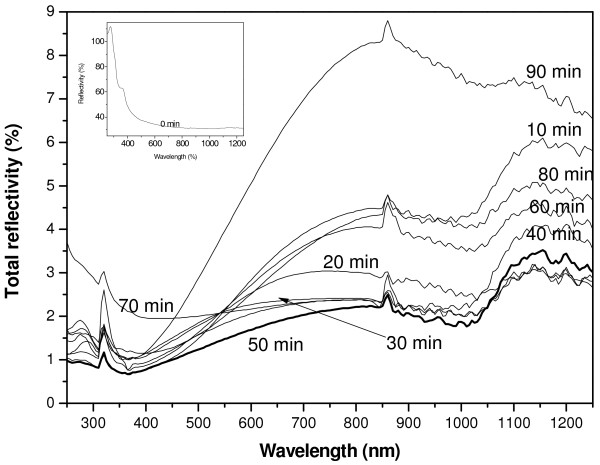
** Total reflectivity spectra of all SiNWs films in the 250 to 1,250 nm wavelength range.** The inset is the total reflectivity of untreated silicon surface.

The total reflectivity in the 250 to 1,250 nm spectral range of formed films has very low values, less than 1% in the UV domain and a maximum of 8% in the visible and near-infrared regions (Figure 4). Generally, radiations with small wavelengths (UV domain) are absorbed at small depth and this absorption depends on the surface morphology. In the case of the non-treated sample, the reflectivity in the UV region is greater than 50% as shown in the inset of Figure 4. However, values of the reflectivity of SiNWs films in the UV domain are in the 0.5% to 1.5% range, which is unusual in silicon, even with texturized morphology and/or porous silicon. This was attributed to the important internal surface area of SiNWs. From curves in Figure 4, we remark that in the fully used spectral range, the SiNWs film elaborated at 50 min has the minimum value of the total reflectivity. The small values of reflectivity are attributed to the multiple reflections of incident photons which are important when the length of SiNWs film is important (38 μm during 50 min).

**Figure 5:**
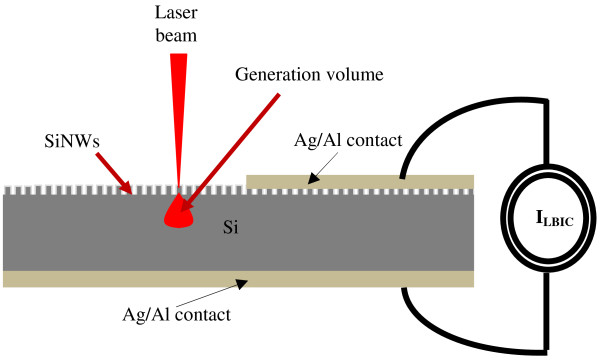
Schematic illustration of the LBIC technique.

**Figure 6:**
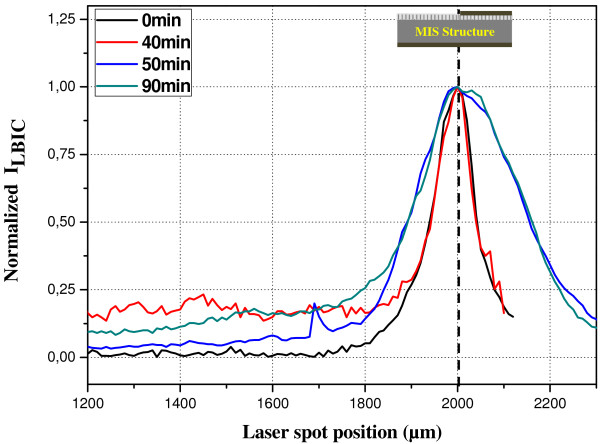
** Typical normalized LBIC profiles.** Vertical dash line corresponds to the metal position on the top of the MIS structure.

For the electronic characterization, we use the LBIC technique at the He-Ne wavelength. A schematic illustration of the LBIC technique is given in Figure 5. LBIC measurements were performed on metal-insulator-semiconductor structures formed on SiNWs films. Typical LBIC profiles are given in Figure 6. We notice that we obtained the same shape of LBIC profiles for samples etched during 30, 50, 60, 70, 80, and 90 min. However, for samples etched during 10, 20, and 40 min, the LBIC profiles have approximately the same shape of that one performed on the MIS diode without SiNWs (0 min). Using the LBIC measurements (*I*_LBIC_), we determine the effective values of *L*. To carry out *L* values, we fit the LBIC theoretical expression given in Equation 1 [16] to the measured LBIC profiles.

(1) $$I_{\mathrm{LBIC}}\propto \frac{exp(-\frac{x}{L})}{x^{\frac{3}{2}}}$$

**Figure 7:**
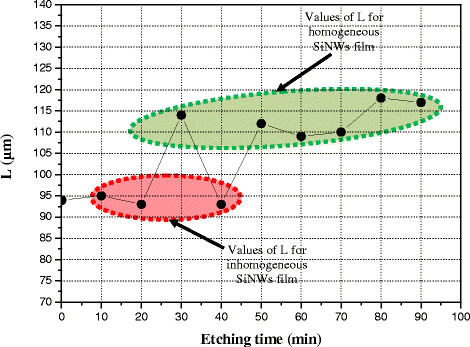
Obtained values of the diffusion length vs. etching time.

Obtained values of the effective diffusion length are plot in Figure 7. We remark that the obtained *L* values can be divided into two domains: red and green regions in Figure 7. The red region corresponds to the *L* values obtained for samples prepared during 0, 10, 20, and 40 min. The green region corresponds to samples prepared at 30, 50, 60, 70, 80, and 90 min. 

**Figure 8:**
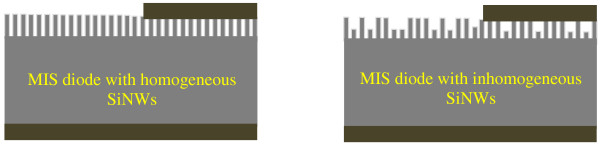
Schematic illustration of the MIS diode when the SiNWs film is homogeneous and not.

To understand why *L* values change from a sample to another, we use the cross-section SEM images of Figure 2 and the total reflectivity of films given in Figure 4. In Figure 2, we remark that durations 10, 20, and 40 min lead to inhomogeneous SiNWs films. However, the cross-section SEM images of samples prepared during 30, 50, 60, 70, 80, and 90 min show homogeneous films. In addition, taken into account that at the used wavelength in the LBIC investigations (He-Ne: 633 nm), the corresponding values of the total reflectivity (Figure 4) cannot explain, for example, why the *L* value of the sample prepared at 90 min is greater than that one prepared at 30 min. Consequently, we attribute these variations not to the total reflectivity, but to the carriers’ trapping at surface defects. For this purpose, we consider the schemes given in Figure 8. Thus, when the MIS diode contains homogeneous SiNWs, a great amount of photo-generated electrons by the laser beam can reach the top metal contact. However, when SiNWs are not homogeneous, surface recombination at small wires reduces the LBIC current value, leading to a decrease in the effective diffusion length.

## Conclusions

In this study, we present a morphological, optical, and electronic study of SiNWs films elaborated at different durations; 10, 20, 30, 40, 50, 60, 70, 80, and 90 min. At some etching durations, a regular structure of formed SiNWs was observed. The SiNWs lengths vary from 21 to 38 μm. We notice a spectacular very low value of the total reflectivity reaching a minimum less than 1% in the 250 to 400 nm and a minimum of 1.5% in the visible domain. From LBIC investigations, we deduce that the homogeneity of the SiNWs film plays a key role on the electronic properties. Hence, we carried out that when the SiNWs film is inhomogeneous, surface recombination of photo-generated carriers can decrease the effective diffusion length.

## Competing interests

The authors declare that they have no competing interests.

## Authors’ contributions

NN prepared samples, MIS diodes, and performed SEM images. She also achieved the reflectivity and LBIC measurements. MAL helped on the preparation of samples, SEM investigations, carrying out the length of SiNWs, and the interpretation of LBIC profiles. Finally, MB supervised the work, did the interpretations, and wrote the text with NN and MAL. All authors read and approved the final manuscript.
